# TNM staging of esophageal cancer using fine-tuned pathology foundation models and multiple instance learning

**DOI:** 10.3389/fonc.2026.1832365

**Published:** 2026-05-29

**Authors:** Junxiang Cao, Jiacheng Wang

**Affiliations:** 1 School of Computer, Jiangsu University of Science and Technology, Zhenjiang, China; 2 Manteia Technology, Co., Ltd., Xiamen, China

**Keywords:** esophageal cancer, foundation model, multiple instance learning, TNM staging, whole slide imaging

## Abstract

**Background:**

Esophageal cancer is a common and aggressive malignancy worldwide. Accurate TNM staging is essential for clinical decision-making and prognosis. While whole slide images (WSIs) offer rich histopathological details for automated staging, their sheer size and complexity make manual analysis subjective and time-consuming. Consequently, advanced deep learning models are critically needed to improve the accuracy and efficiency of WSI-based TNM staging.

**Methods:**

We propose a novel deep learning approach that integrates a fine-tuned pathology foundation model within a Multiple Instance Learning (MIL) framework for automated TNM staging from WSIs. To boost predictive accuracy, our model is enhanced with a feature attention mechanism to pinpoint critical tumor areas and an adaptive layer for effective multi-scale feature fusion. Furthermore, we developed a class-weighted cross-entropy loss function to address pronounced TNM stage imbalance, targeting composite pathological stage classification as a single multi-class prediction task rather than separate T, N, and M components. Performance was rigorously evaluated using three-fold cross-validation and benchmarked against state-of-the-art methods.

**Results:**

Our model achieved outstanding performance on the TCGA-ESCA dataset, outperforming existing methods in accuracy, macro F1-score, and AUC. These results confirm the model’s effectiveness. Visualization analysis further showed that the model accurately localizes tumor regions and their boundaries, increasing the interpretability and reliability of its predictions.

**Conclusion:**

Our study validates the potential of a WSI-based deep learning approach for accurate prediction of esophageal cancer’s TNM stage. This tool has the potential to augment pathological diagnosis, refine patient risk assessment, and ultimately guide better treatment strategies.

## 1 Introduction

Esophageal cancer (ESCA) is a prevalent and highly lethal malignancy of the digestive tract worldwide, and early diagnosis together with accurate staging is critical for improving patient outcomes (1, 2). The two major histological subtypes of ESCA, squamous cell carcinoma (ESCC) and adenocarcinoma (AC), differ markedly in pathogenesis, epidemiological characteristics, and prognosis, and they also require distinct therapeutic strategies (3). Currently, the TNM staging system is a widely accepted standard for treatment decision-making and prognostic evaluation in ESCA, providing essential guidance for clinical management. This system classifies patients according to pathological parameters including the depth of primary tumor invasion (T), the extent of regional lymph node involvement (N), and the presence of distant metastasis (M), thereby enabling stratified prognostic assessment and supporting personalized treatment planning (4, 5).

In recent years, with the continuous advancement of digital pathology, whole slide imaging (WSI) has become increasingly prevalent in clinical pathological diagnosis. By enabling high-resolution scanning of entire tissue sections, WSI allows for comprehensive digital storage and visualization of slides, enabling pathologists to freely zoom, navigate, and examine histological structures from multiple perspectives at a computer workstation. This capability has markedly improved both the efficiency and accuracy of diagnosis (6–8). Furthermore, WSI not only overcomes the limitations of conventional optical microscopy in terms of field of view and information accessibility but also expands the scope of pathology applications, providing robust support for telepathology, pathology education, case archiving, and the integration of artificial intelligence (AI) into diagnostic practice. Multiple studies have demonstrated that WSI achieves a high level of diagnostic concordance with traditional glass slides across a wide range of tumors. Moreover, in cancers with complex histological architecture and stringent staging requirements, such as ESCA, WSI offers a more comprehensive and objective representation of spatial distribution and infiltration patterns (9).

In TNM staging of ESCA, WSI provides a more accurate and visually intuitive evaluation of the depth of primary tumor invasion, extent of lymph node involvement, and presence of distant metastasis. For instance, WSI enables continuous examination of entire tissue sections, facilitating the detection of small lymph node metastases and early tumor infiltration, thereby reducing the risk of missed diagnoses and misclassification (10, 11). Moreover, the digital nature of WSI offers a rich data foundation for the development of AI algorithms and automated staging assessment, holding significant potential to advance the standardization and intelligence of ESCA pathological staging.

However, the extremely large data scale of WSI and the limited granularity of available annotations pose significant challenges for analysis. Individual slides often contain hundreds of millions of pixels, while annotations are typically provided only at the slide level, lacking detailed labels for specific tissue regions. This limitation hinders the direct application of conventional supervised learning methods. In this context, the integration of digital pathology and AI has driven rapid advances in computational pathology (12, 13). Deep learning (DL) models, in particular, have demonstrated remarkable feature learning capabilities, enabling the automatic extraction of complex histological patterns, many of which surpass the visual perception of human observers. To address these challenges, the multiple instance learning (MIL) framework has been widely adopted (14, 15). MIL aggregates information from local image patches to generate slide-level predictions, allowing efficient processing of entire WSI without dense annotations. Furthermore, the incorporation of attention mechanisms enables the model to focus on diagnostically relevant regions within the tumor microenvironment, thereby significantly enhancing the accuracy and reliability of feature extraction and prediction (16–18).

Recent advances in imaging technology and computer-aided diagnosis (CAD) are further transforming the landscape of esophageal cancer detection. Notably, hyperspectral imaging (HSI) combined with deep learning has demonstrated promising results for early esophageal cancer detection, with YOLO-based hyperspectral models achieving high sensitivity for mucosal lesions that may be missed under conventional white-light endoscopy (19, 20). These complementary modalities highlight a broader paradigm shift toward AI-assisted, multimodal esophageal diagnostics. Our work contributes to this landscape by addressing a distinct and underexplored challenge: automated pathological TNM staging directly from WSIs of resected specimens, which provides a critical layer of objective histopathological assessment independent of endoscopic or radiological workup.

Building on the aforementioned background, this study aims to develop a novel MIL-based DL model that leverages only WSIs of the primary tumor to composite overall pathological TNM stage (Stage I–IV as a single multi-class classification task). To enhance the model’s predictive capability, we incorporate a feature attention mechanism to strengthen the representation of diagnostically critical regions, enabling the network to focus on histological areas most relevant to tumor staging. Additionally, an adaptive layer is employed for multi-scale feature fusion, allowing the integration of information across different spatial resolutions and improving prediction performance across various staging categories. By combining these strategies, the model achieves improved accuracy, robustness, and interpretability.

## 2 Related work

AI has rapidly advanced in digital pathology, particularly in DL applications that span image segmentation, classification, prognosis prediction, and treatment response evaluation (21–24). Relevant studies across various cancers have demonstrated clinical value, such as subtype differentiation in lung cancer using pathology slides (25), prognosis prediction in colorectal cancer (26), precision treatment exploration in hepatocellular carcinoma (27, 28), and Gleason grading prediction in prostate cancer biopsies (29). These achievements highlight the broad applicability and clinical potential of DL in pathological imaging.

In the field of ESCA, DL research has primarily focused on endoscopic image diagnostics, especially early detection and grading of Barrett’s esophagus (BE) and esophageal squamous cell carcinoma. For instance, Gong et al. (30) developed a model based on HDWLE images, achieving an accuracy of 93.9% in multi-center external testing; Tang et al. (31) introduced a real-time DCNN system, which significantly outperforms expert endoscopists in sensitivity and NPV; Cai et al. (32) developed a CAD system that exceeds the sensitivity and NPV of endoscopists at various levels, effectively improving overall diagnostic performance in clinical practice; Li et al. (33) demonstrated that CAD-NBI outperforms CAD-WLI in accuracy and specificity, especially among mid-level and junior endoscopists.

In addition to conventional endoscopic detection, some studies have extended DL to BE detection and quantitative analysis. Pan et al. (34) employed a fully convolutional network (FCN) to segment the gastroesophageal junction (GEJ) and squamocolumnar junction (SCJ), assisting BE detection; Tsai et al. (35) used EfficientNetV2B2 to build a CAD system that achieves over 94% accuracy and sensitivity on NBI images; Wu et al. (36) developed the ELNet system, which integrates classification and segmentation modules, outperforming existing methods in sensitivity and accuracy; Ali et al. (37) developed a DL-based 3D reconstruction system capable of automatically quantifying BE length and area, accurately extracting C&M scores. These studies not only demonstrate the potential of DL for automatic BE detection but also offer new approaches for quantification and standardization.

Moreover, research has extended to other modalities such as CT and pathology slides. Markowetz et al. (38) demonstrated the efficient diagnosis of BE using Cytosponge-TFF3 detection on pathology slides, reducing the pathologist’s workload by 57%; Bouzid et al. (39) proposed the BE-TransMIL model, which showed excellent generalization capability on H&E and TFF3-stained slides, with external validation AUROC exceeding 87%; in CT imaging, efforts have been made to predict chemoradiation therapy (CRT) responses in ESCA patients using DL models (30, 40). However, there is still a lack of DL research on WSI for differentiating ESCC and AC morphological features, or predicting CRT treatment responses, which remains an area for future breakthroughs.

Despite the significant potential of DL for early diagnosis and staging of ESCA, most research has focused on endoscopic images and other imaging modalities. In contrast, automated TNM staging based on WSI is still in its infancy and faces many challenges. Therefore, automated ESCA TNM staging prediction based on pathology slides holds significant clinical promise and represents a new technological breakthrough for future intelligent pathology image analysis and personalized treatment. The proposed research aims to lay a crucial foundation for precise diagnosis, clinical decision-making, and individualized treatment of ESCA, and provides a model that can be adapted for the pathological analysis of other cancer types.

## 3 Materials and methods

### 3.1 Patients and data collection

This study analyzes the ESCA dataset from The Cancer Genome Atlas (TCGA). The TCGA-ESCA cohort includes 151 pathologically confirmed ESCA patients, with a total of 151 H&E stained WSIs from these patients. All data were publicly obtained from the TCGA official data portal (https://portal.gdc.cancer.gov/).

This study followed the criteria outlined below for patient selection:

Inclusion criteria:

1. Pathologically diagnosed with ESCA.
2. Availability of H&E WSIs.
3. Complete American Joint Committee on Cancer (AJCC) TNM staging data and survival follow-up information.

Exclusion criteria:

1. Poor tissue slide quality (e.g., excessive fading, missing critical areas, severe folding, or artificial artifacts such as knife marks), which would affect subsequent morphological analysis.
2. Incomplete clinical pathological information (e.g., missing critical staging information, unknown survival status, or insufficient follow-up time of less than 30 days).
3. Specimens obtained after neoadjuvant therapy and surgical resection (to avoid interference from treatment-related morphological changes in the analysis).

After applying these criteria, a total of 128 patients were included in the subsequent analysis. All clinical data acquisition and data usage for the selected patients were in compliance with the TCGA data use guidelines and relevant ethical regulations. This study exclusively utilized retrospective, de-identified human pathological and clinical data from the publicly accessible TCGA repository; no direct patient contact or prospective data collection was performed. Use of TCGA data is governed by the TCGA Data Use Certification Agreement, and no additional institutional review board (IRB) approval was required for this publicly available, de-identified dataset. All WSIs used in this study are H&E-stained slides derived from primary esophageal resection specimens; no lymph node dissection slides or metastatic site specimens were included. The final cohort comprised 80 esophageal squamous cell carcinoma (ESCC) and 45 adenocarcinoma (AC) cases (3 not reported). TNM stage distribution was as follows: Stage I, n=14 (10.9%); Stage II, n=64 (50.0%); Stage III, n=45 (35.2%); Stage IV, n=5 (3.9%). The cohort included 109 male and 19 female patients, with a mean age at diagnosis of 60.1 ± 10.6 years (range 36–86). During 3-fold cross-validation, stratified splitting was employed to maintain consistent stage and histological subtype proportions across folds. Patient characteristics are summarized in **Supplementary Table 1**.

### 3.2 Data preprocessing and enhancement

In this study, we adopted the CLAM (Classification of Large-scale Pathology Whole Slide Images) standard pipeline to process and analyze WSI of ESCA. This pipeline provides an efficient solution for handling large-scale pathology image data, ensuring high-quality feature extraction and model training.

First, we used the OpenSlide library (v. 1.3.1) to load WSI files and parse their multi-resolution pyramid structure. All subsequent processing was conducted at a 40x magnification level to ensure sufficient tissue detail. Prior to cutting image patches, we performed background filtering to remove non-tissue areas, blank regions, and artifacts, ensuring that the extracted image patches contained valid tissue information. Next, fixed-size image patches (224×224 pixels) were extracted from the cleaned pathology slides. These image patches represent localized tissue information, and together, they form the representation of the entire slide. To mitigate color variations between samples, we performed color normalization on the extracted image patches. The Reinhard color normalization method was applied to adjust all slides to a unified color standard. This process allowed the model to focus more on tissue structural features rather than color variations, thereby improving its generalization capability. To address the long-tail problem, we employed a 3-fold cross-validation strategy for data partitioning.

### 3.3 MIL model construction

**Figure 1:**
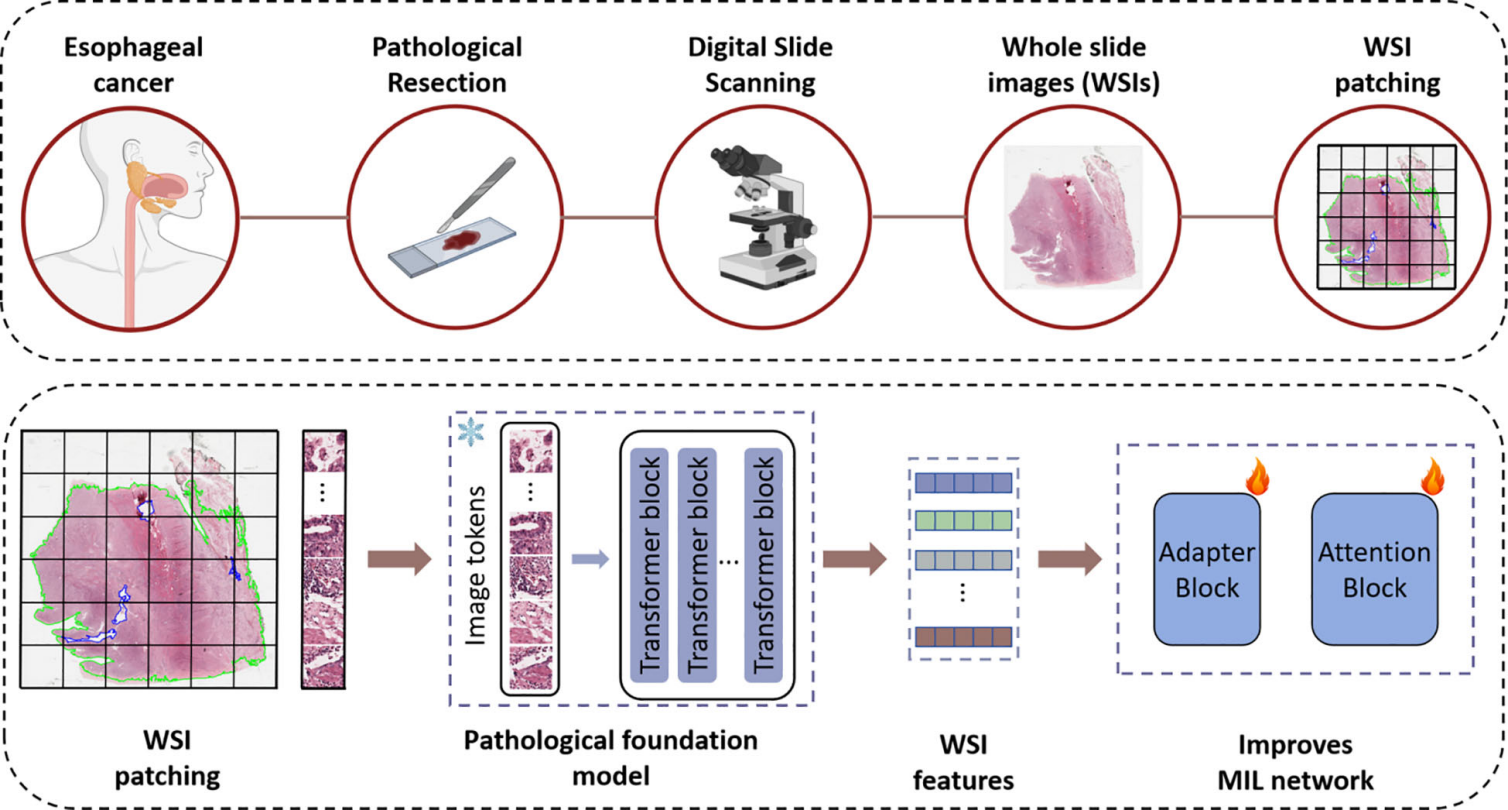
Architecture of the proposed model.

To address the high dimensionality and strong noise inherent in directly modeling WSIs, this study developed an enhanced slide-level prediction model based on the classical MIL framework. The overall architecture consists of three core stages: feature extraction, instance feature aggregation, and slide-level classification. The model structure is shown in **Figure 1**. In the feature extraction stage, preprocessed image patches are encoded using a pretrained universal pathology feature encoder to obtain high-level semantic features with dimensionality d. Compared with training CNNs from scratch, leveraging a large pretrained model effectively enhances feature generalization and mitigates overfitting in small-sample scenarios. During the feature aggregation stage, all instance features from a single WSI are input into an attention-based MIL module. This module learns discriminative attention weights, assigning higher importance to key tumor regions while preserving global information, thereby emphasizing regions relevant to cancer staging. Finally, the slide-level embedding generated by the MIL module is passed through a fully connected classifier to predict multi-class labels. Unlike conventional mean or max pooling, the attention-driven aggregation employed in this study allows finer modeling of inter-instance variability and improves the recognition of long-tail categories.

**Figure 2:**
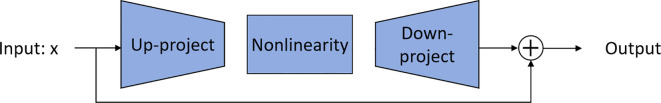
Architecture of the adapter for residual-style feature enhancement.


Accurately identifying key information relevant to cancer staging from noisy instances in the MIL aggregation module is critical for model performance. In this study, we introduce a combined attention and adapter structure within the aggregation module to achieve adaptive feature refinement and efficient modeling of important regions. **Figure 2** shows the details of the adapter module used for residual-style enhancement. First, the input instance features x are mapped to a latent space of dimension L through a linear transformation followed by a nonlinear activation. Subsequently, a residual-style enhancement is applied via the adapter module:

$$\text{Adapter}\left(x\right)=x+W_{up}\sigma \left(W_{down}x\right)$$

Here, W_down_ and W_up_ denote the linear layers for down-projection and up-projection, respectively, and σ represents the nonlinear activation function. This adapter module enables lightweight nonlinear adjustments while maintaining the stability of the original features, thereby enhancing the model’s ability to capture fine-grained differences.


$$A=\text{softmax}\left(W_{a}\text{tanh}\left(Vx^{\text{T}}\right)⊙\text{σ}\left(Ux^{\text{T}}\right)\right)$$

**Figure 3:**
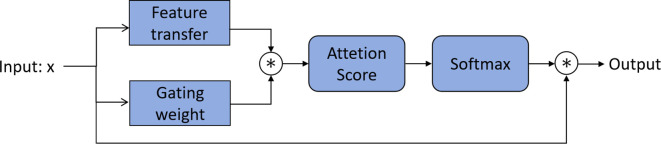
Architecture of the gated attention.


Regarding the attention mechanism, the network uses Gated Attention. **Figure 3** shows the details of the Gated Attention. The attention weights are computed as follows:

$$M=\sum_{i=1}^{N}A_{i}x_{i}$$

This architecture not only enhances the model’s sensitivity to critical instances through the attention mechanism but also increases the flexibility of feature representations via the Adapter module, enabling the model to better handle the heterogeneity and long-tail distribution inherent in pathology WSIs. Compared with conventional attention-based MIL, the proposed Adapter-enhanced structure demonstrates improved robustness in feature representation and model generalization.

During the training of the multi-instance learning model, the TNM stage distribution of ESCA exhibits a pronounced long-tail pattern, with substantial differences in sample sizes across stages. To mitigate the adverse effects of class imbalance on model training, this study employs a weighted cross-entropy loss function for optimization. Specifically, for each class *c* with *n_c_* samples, the class weight is calculated as:


$$w_{c}=\frac{1}{\text{log}\left(1+n_{c}\;\right)}$$

Here, the form of log(1 + *n_c_*) allows the model to assign higher training attention to underrepresented classes while avoiding excessively large or small weights. The weighted cross-entropy loss function is then defined as:


$${ℒ}_{CE}=-\sum_{c=1}^{C}w_{c}y_{c}\text{log}\left({\hat{y}}_{c}\right)$$

Here, *y_c_* denotes the one-hot encoding of the true label, ${\hat{y}}_{c}$ represents the predicted probability from the model, and *C* is the total number of classes. This weighting strategy enables the model to learn more effectively from underrepresented classes during training, thereby improving the predictive performance for rare TNM stages, while maintaining stable performance for the majority classes.

### 3.4 Model development and validation

In the feature extraction stage, image patches were encoded using a pretrained UNI (41) to obtain high-level semantic features capturing cellular morphology, tissue architecture, and microenvironment information. These features enhance generalization in small-sample scenarios and are subsequently aggregated via the MIL module for slide-level prediction. In addition, we also employed CONCH (42) for feature extraction as a comparative baseline. For data splitting, this study employed 3-fold cross-validation with stratified sampling to preserve TNM stage and histological subtype (ESCC/AC) distributions across folds to ensure that each patient participates in both training and testing, thereby enhancing the robustness of model performance. In each fold, the dataset was divided into training and validation sets, and the final results were obtained by averaging the performance metrics across the three folds. Implementation details include using the PyTorch framework on an NVIDIA A100 GPU (48 GB). The Adam optimizer was employed with an initial learning rate of 0.0001, dynamically adjusted using a cosine annealing scheduler. Since the MIL framework trains at the slide level, the batch size was set to 1, with 50 training epochs per fold. For each fold, the model achieving the best validation performance was saved. This training strategy ensures both the generalizability and stability of the model in complex pathological scenarios.

### 3.5 Statistical analyses

All statistical analyses in this study were conducted using Python (v3.10). Model performance was assessed using metrics including Accuracy (ACC), F1-score, and the Area Under the Curve (AUC) of the multi-class ROC. For cross-validation results, the mean and standard deviation across folds were reported to evaluate model stability. Multi-class confusion matrices were employed to visualize predictive performance across different TNM stages.

## 4 Results

### 4.1 Comparison of MIL models

The **Table 1** presents the classification performance of various MIL models using two types of feature extractors, CONCH and UNI, with metrics including ACC, AUC, and F1 score. Overall, both the choice of feature extractor and the MIL model significantly affect performance. When using Conch features, DS_MIL outperforms other traditional MIL models in ACC, yet its F1 score remains lower than that of our proposed method, indicating that conventional approaches may struggle with imbalanced samples or identifying key instances. Our method achieves an ACC of 71.43%, AUC of 91.70%, and F1 of 74.28% on Conch features, demonstrating superior performance in both accuracy and comprehensive metrics, reflecting its ability to capture important instances and integrate global information effectively.

**Table 1:** Performance comparison of different MIL-based models on CONCH and UNI feature extractors.

Feature 3xtractor	Models	ACC(%)	AUC(%)	F1(%)
CONCH	AB_MIL	64.28	89.33	44.59
	DS_MIL	71.43	93.69	68.94
	CLAM_MIL	59.52	61.31	59.52
	Trans_MIL	61.90	89.18	44.32
	Ours	71.43	91.70	74.28
UNI	AB_MIL	73.81	87.10	72.64
	DS_MIL	76.19	92.52	79.76
	CLAM_MIL	76.19	90.34	76.19
	Trans_MIL	66.67	88.00	66.67
	Ours	78.57	94.05	81.36

Metrics include ACC, AUC, and F1 score. “Ours” denotes the proposed method, which achieves the highest overall performance across both feature extractors.

With UNI features, all models perform better compared to Conch, suggesting that UNI features provide richer discriminative information. While DS_MIL and CLAM_MIL achieve an ACC of 76.19%, our proposed method attains the highest values across ACC (78.57%), AUC (94.05%), and F1 score (81.36%), highlighting its robustness and generalization capability across different feature spaces. Overall, regardless of the feature extractor, our method consistently captures critical pathological information and improves classification performance, showing clear advantages over existing MIL approaches.

### 4.2 Ablation studies

**Table 2** summarizes the performance of various models using two different feature extractors, CONCH and UNI, evaluated in terms of ACC, AUC, and F1 score. For the CONCH feature extractor, the baseline model achieves an ACC of 64.28%, an AUC of 89.33%, and an F1 score of 44.59%. Introducing the adapter module substantially improves all metrics, with ACC rising to 69.05%, AUC to 94.29%, and F1 to 74.23%. The Attention module enhances ACC and F1 moderately but slightly decreases AUC. Our proposed method outperforms the baseline and attention variants in ACC and F1, achieving 71.43%, 91.70%, and 74.28%, respectively.

**Table 2:** Ablation experiments evaluating the contribution of different modules in the proposed framework.

Feature 3xtractor	Models	ACC(%)	AUC(%)	F1(%)
CONCH	Baseline	64.28	89.33	44.59
	+Adapter	69.05	94.29	74.23
	+Attention	69.05	88.78	67.86
	Ours	71.43	91.70	74.28
UNI	Baseline	69.05	88.78	67.86
	+Adapter	76.19	91.84	81.40
	+Attention	73.81	94.24	74.60
	Ours	78.57	94.05	81.36

For the UNI feature extractor, the baseline model already achieves higher performance (ACC 69.05%, AUC 88.78%, F1 67.86%) compared with CONCH. Incorporating the Adapter module further boosts all metrics, reaching ACC 76.19%, AUC 91.84%, and F1 81.40%. The Attention module achieves a high AUC of 94.24% but a relatively lower F1 score. Our approach consistently maintains strong performance across all metrics, achieving ACC 78.57%, AUC 94.05%, and F1 81.36%. Overall, these results demonstrate that the proposed method achieves superior or competitive performance across different feature extractors, particularly in terms of ACC and F1, indicating its effectiveness in capturing discriminative features for the classification task.

### 4.3 Explainability

**Figure 4:**
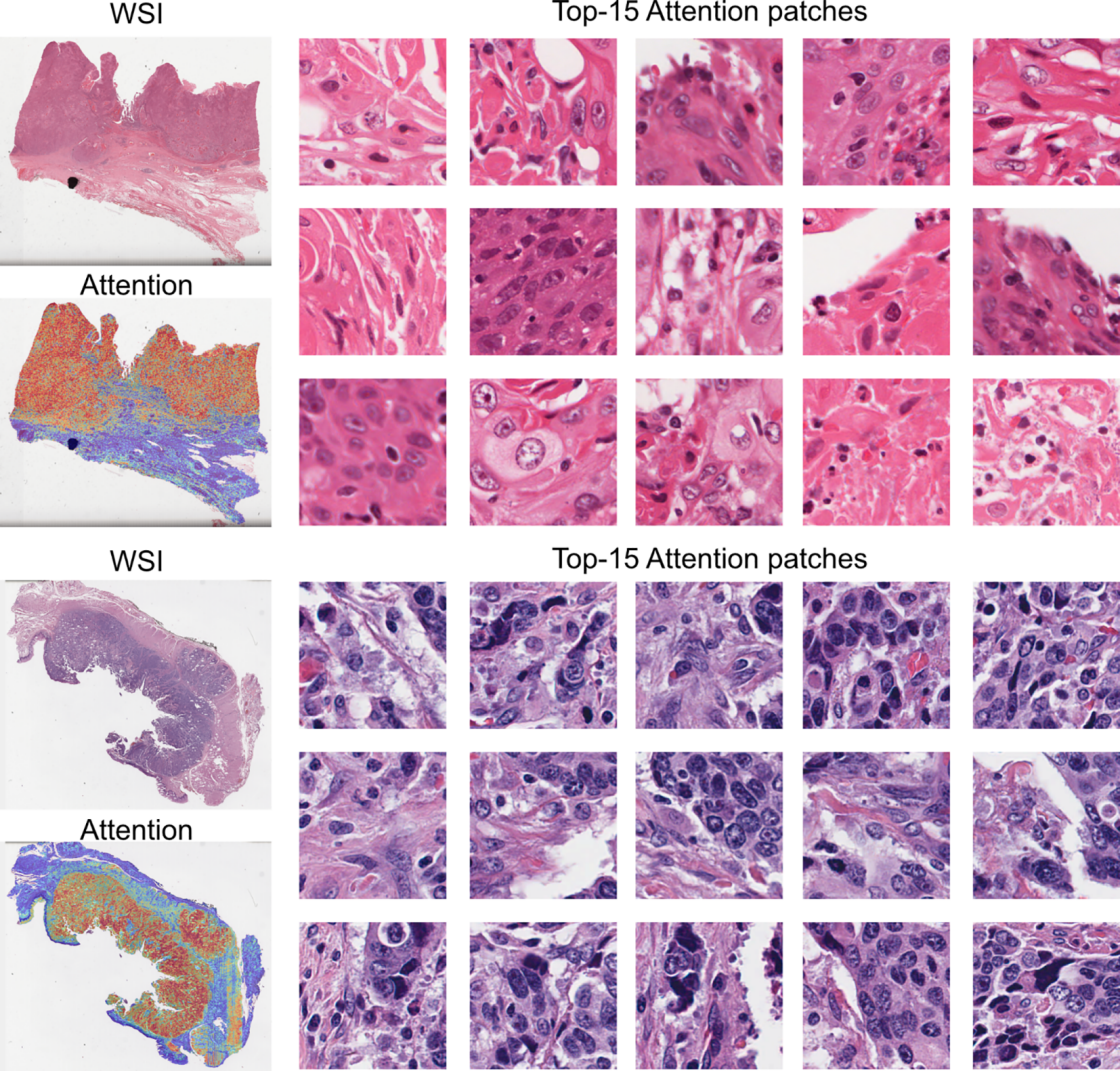
Visualization of attention-based patch selection in ESCA WSIs.

To further interpret the model’s predictions and evaluate its focus on diagnostically relevant regions, we visualized the attention distribution across WSIs. As shown in **Figure 4**, the attention heatmaps indicate that the model assigns higher weights to regions with dense tumor cells, nuclear atypia, and structural abnormalities. The top-15 attention patches extracted from each WSI correspond to areas critical for TNM staging, such as regions with invasive tumor fronts or high-grade dysplasia. These visualizations confirm that the MIL-based model not only integrates information from the entire slide but also effectively identifies the most informative tissue regions, enhancing interpretability and clinical relevance of the predictions.

## 5 Discussion

In this study, we successfully developed a deep learning model based on WSIs that leverages a MIL framework to predict TNM staging of ESCA. The proposed model demonstrates that pathology-driven AI can provide accurate, slice-level predictions of overall TNM stage, highlighting the feasibility of automated histopathological staging in clinical practice.

A key advantage of our model lies in its ability to focus on critical morphological regions that are closely associated with tumor invasion and metastasis. Attention-driven aggregation enables the model to highlight regions that may reflect biological behaviors underlying disease progression, including lymphovascular invasion and submucosal spread. Compared with conventional TNM staging, which relies on manual pathological assessment, our model captures subtle morphological cues across the entire slide that are often overlooked. Similar approaches in other cancers, such as colorectal, lung, and breast cancer, have shown that MIL-based models can effectively link histopathological features with clinically relevant outcomes, demonstrating the translational potential of this methodology. Although N and M status cannot be directly observed on primary tumor WSIs, established histological surrogates of metastatic potential — including lymphovascular invasion, tumor budding, invasion depth, and high-grade nuclear atypia — are spatially encoded within the primary resection specimen and are precisely the type of patch-level signal that attention-based MIL is designed to aggregate. The composite pathological TNM stage thus serves as a valid, biologically grounded supervisory signal, and the model learns to approximate it from primary tumor morphology rather than from direct N or M observation. This paradigm is consistent with WSI-based prediction of nodal status and molecular subtypes from primary tumor histology in colorectal and lung cancer (24, 43). We acknowledge, however, that Stage III vs. IV discrimination — where M1 is the sole differentiator — represents the biological ceiling of this approach, and has informed our decision to frame the present results as proof-of-concept performance.

Clinically, WSI significantly improves the detection of small lesions. The prognosis of ESCA is closely associated with lymph node metastasis, yet the identification of micrometastases under conventional microscopy heavily relies on the pathologist’s experience and attention, posing a risk of missed diagnoses. The high-resolution digital images provided by WSI offer a solid foundation for the development of artificial intelligence algorithms, facilitating automated and standardized pathological staging. For instance, deep learning models have demonstrated excellent performance in identifying tumor regions and quantifying invasion depth, with efficiency and reproducibility far surpassing manual evaluation. This not only alleviates pathologists from repetitive tasks, allowing them to focus on challenging cases, but also optimizes the overall diagnostic workflow, enhancing both clinical efficiency and accuracy.

Despite these promising results, this study has several limitations. First, the dataset was derived solely from TCGA, which may limit the generalizability of the model due to sample selection biases, limited histological diversity, and the absence of inter-institutional staining and scanner variability. The present findings should therefore be interpreted as proof-of-concept performance rather than clinical readiness; external multi-institutional validation is essential before translational deployment and is planned as a primary objective of future work. Second, the relatively small cohort (n=128) and pronounced class imbalance across TNM stages—particularly the underrepresentation of Stage I and Stage IV cases—may constrain the model’s learning of rare-stage-specific features, despite the use of class-weighted loss and stratified cross-validation. Expanding the dataset through additional public repositories (e.g., CPTAC-ESCA) and prospective multi-center data collection will be prioritized. Third, while attention heatmaps provide qualitative interpretability, quantitative validation against pathologist-annotated regions of interest (e.g., invasion fronts, lymphovascular invasion foci) is lacking; future work will incorporate such expert-guided evaluation to confirm the biological relevance of model-highlighted regions. Future work will focus on expanding the dataset to multiple centers to improve generalization and robustness. It is important to emphasize that the proposed WSI-based model is intended to augment, not replace, conventional radiological staging workup including computed tomography (CT) and positron emission tomography (PET-CT). While our approach provides complementary pathological staging information from resected primary tumor slides, it does not assess distant metastatic burden or pre-operative nodal status, which require dedicated imaging modalities. The clinical value of this tool lies in its ability to provide an objective, reproducible, and automated corroboration of pathological stage from histology alone, supporting multidisciplinary tumor board review and informing post-operative prognostic assessment. Moreover, integrating multimodal information—including radiological imaging, pathological slides, and genomic data—could further enhance predictive performance and provide a more comprehensive understanding of tumor biology, paving the way for more precise and individualized clinical decision-making.

## 6 Conclusion

In this study, we developed a deep learning model based on WSIs using a MIL framework to automatically predict the overall TNM stage of ESCA. By integrating attention mechanisms with adapter modules, the model effectively focuses on critical pathological regions, enhances recognition of underrepresented stage categories, and improves overall prediction accuracy and robustness. This work provides a practical approach for automated pathological staging of ESCA and lays the foundation for future multimodal integration, personalized treatment decision-making, and intelligent pathology analysis.

## Data Availability

The original contributions presented in the study are included in the article/**Supplementary Material**. Further inquiries can be directed to the corresponding author.

## Supplementary material

Supplementary Table 1 Patient characteristics and TNM stage distribution (n = 128).
