# Supplementary material for: TNM staging of esophageal cancer using fine-tuned pathology foundation models and multiple instance learning
Source: Front Oncol. 2026 May 29;16:1832365. doi: 10.3389/fonc.2026.1832365 (PMC13259655; doi:10.3389/fonc.2026.1832365)
Supplement: Supplementary Table 1 — Patient characteristics and TNM stage distribution (n = 128). [file DataSheet1.pdf]

Supplementary Table S1. Patient Characteristics and TNM Stage Distribution (n = 128)

Characteristic	Category	n (%)
**TNM Stage**		
	Stage I	14 (10.9%)
	Stage II	64 (50.0%)
	Stage III	45 (35.2%)
	Stage IV	5 (3.9%)
**Histological Subtype**		
	ESCC	80 (62.5%)
	AC	48 (37.5%)
**Sex**		
	Male	109 (85.2%)
	Female	19 (14.8%)
**Age at Diagnosis (years)**		
	Mean ± SD	60.1 ± 10.6
	Range	36–86
